# Precautions in Bathing

**Published:** 1889-01

**Authors:** 


### PRECAUTIONS IN BATHING.

The bathing season, though not yet advanced, has already been marked by the levy of that fatal tribute which year by year is exacted of the ignorant and the indiscreet. The recent death by drowning of a young man in the public baths at Poplar suggests one cause of accident which is too apt to be overlooked. The deceased had entered the water soon after partaking of a hearty meal, and the fatal result was attributed to cerebral congestion due to sudden immersion at such a time. What may have been the particular appearances observed after death in this case we have no means of judging, but it may be well to consider shortly some reasons why the practice of bathing soon after meals is justly condemned. Effusion of blood in or upon the brain, when it occurs in such cases as that already referred to, is probably not a primary cause of mischief, but rather a consequence founded on other circulatory and nervous disturbances. It is an evidence of eclampsia, and the physiological basis upon which this is founded consists in that inward diversion of blood toward the alimentary tract which characterizes normal digestion, the other tissues, notably the brain, being at the same time proportionally anæmic, and the action of heart, and lungs impeded by a distended stomach. A natural result of cold immersion at this stage is to encourage or induce a tendency to syncope, to concentrate surface blood still more about the central organs, including the heart, which especially, if at all unequal to its duties, labors ineffectually to readjust the blood pressure, and finally succumbs with lungs and venous system engorged by passive congestion. It is as if an enemy occupied the outworks of a fortress left for a time unguarded, and forthwith paralyzed the resistance of the citadel. It is best, therefore, to wait for at least an hour and a half or two hours after a good meal before bathing. Another danger to be avoided is that of a cramp. This is particularly apt to occur after severe exercise or long immersion. The effect of cold being to prolong the contraction, while exhaustion lowers both the power and the elastic recoil of muscle, it is evident that we have in a combination of these forces all that is required for the production of this dangerous condition. The obvious warning implied in these remarks requires no further admonition to impress the fact that the bather in cold water must be economical of time and free from any appreciable signs of muscular exhaustion.—*Lancet.*

